# Randomized, double-blind, noninferiority study of diclofenac diethylamine 2.32% gel applied twice daily versus diclofenac diethylamine 1.16% gel applied four times daily in patients with acute ankle sprain

**DOI:** 10.1186/s12891-022-06077-z

**Published:** 2022-12-24

**Authors:** Feng Yin, Jinzhong Ma, Haijun Xiao, Rongguang Ao, Fengqi Zhang, Wencui Li, Wei Wang, Peter Zeng, Tracy Lu, Frédérique Bariguian Revel, Mako Araga, Shiva Patel, Sebastian Moreira, Junfei Zhang, Weibin Zhang

**Affiliations:** 1 Shanghai East Hospital, Shanghai, China; 2 Shanghai General Hospital, Shanghai, China; 3 Shanghai Fengxian Central Hospital, Shanghai, China; 4 Shanghai Pudong Hospital, Shanghai, China; 5 The Third Hospital of Hebei Medical University, Shijiazhuang, China; 6 Shenzhen Second People’s Hospital, Shenzhen, China; 7 Shenyang Orthopedic Hospital, Shenyang, China; 8 GSK China Consumer Healthcare, Shanghai, China; 9 GSK Consumer Healthcare SARL, Nyon, Switzerland; 10 GSK Consumer Healthcare, Warren, NJ USA; 11 Ruijin Hospital, Shanghai Jiaotong University School of Medicine, Shanghai, China

**Keywords:** Diclofenac diethylamine gel, Ankle injury, Sprain, Analogue pain scale

## Abstract

**Background:**

Diclofenac diethylamine (DDEA) gel has demonstrated efficacy for treatment of ankle sprains in both the 1.16% four-times-daily (QID) and 2.32% twice-daily (BID) formulations. The objective of this study was to compare, for the first time, the efficacy of DDEA 2.32% gel BID and DDEA 1.16% gel QID.

**Methods:**

This was a phase 3, randomized, double-blind, multicenter, active-controlled, parallel-group study conducted in China from October 2019 to November 2020, designed to determine the noninferiority of DDEA 2.32% gel BID relative to DDEA 1.16% gel QID for treatment of grade I–II ankle sprain. At study entry, patients must have had pain on movement (POM) ≥50 mm on a 100-mm visual analogue scale (VAS), and not received any pain medication. The primary efficacy endpoint was the noninferiority of DDEA 2.32% gel BID vs DDEA 1.16% gel QID for POM as assessed by the patient using the 100-mm VAS, conducted on day 5. Secondary endpoints included measures of ankle tenderness, joint function, swelling, and patient-reported pain intensity and pain relief.

**Results:**

A total of 302 patients were randomized and 95.4% completed the study. The mean (SD) change in POM from baseline to day 5 using the 100-mm VAS was − 42.8 mm (19.7 mm) with DDEA 2.32% gel BID and − 43.1 mm (18.1 mm) with DDEA 1.16% gel QID for the per-protocol population. The least squares mean difference (DDEA gel 2.32% – DDEA gel 1.16%) at this timepoint was 1.11 mm (95% CI − 3.00, 5.22; *P* = 0.595), and the upper limit (5.22 mm) of the 95% CI was less than the noninferiority margin of 13 mm, demonstrating that DDEA 2.32% gel BID was noninferior to DDEA 1.16% gel QID. Similar trends were seen for the secondary efficacy endpoints. There was no significant difference in the incidence of treatment-emergent adverse events or adverse events adjudicated as being treatment related. All treatment-related adverse events were dermatological; one patient discontinued from the DDEA 2.32% gel BID arm due to application-site inflammation.

**Conclusions:**

DDEA 2.32% gel BID offers a convenient alternative to DDEA 1.16% gel QID, with similar pain reduction and relief, anti-inflammatory effects, and tolerability.

**Trial registration:**

NCT04052620.

## Introduction

Ankle injuries are among the most common incurred by athletes, accounting for 10 to 30% of all sports injuries [1]. Ankle sprains can cause inflammation, which is associated with pain, edema, hyperalgesia, and erythema [2]. Typical therapy for such sprains includes rest, ice, compression, and elevation (RICE), as well as nonsteroidal anti-inflammatory drugs (NSAIDs) [3, 4].

Diclofenac is an NSAID commonly used to treat ankle sprains and is effective in the relief of pain and inflammation. Diclofenac also is used as an antipyretic and is effective in reducing body temperature due to fever. Its mechanism of action is via suppression of prostaglandin synthesis caused by inhibition of the enzyme cyclooxygenase 2 (COX-2) [5]. Diclofenac diethylamine (DDEA) gel is a topical formulation indicated for the relief of pain, inflammation, and swelling in patients with soft tissue injuries, including trauma of the tendons, ligaments, muscles, and joints due to sprains, strains, bruises, and backache. It is also indicated for localized forms of soft tissue rheumatism, tendonitis (eg, tennis elbow), bursitis, shoulder-hand syndrome, periarthropathy, and nonserious arthritis of the knee or fingers. DDEA gel is available in 2 strengths, DDEA 1.16% gel and (in some countries) DDEA 2.32% gel. The efficacy and safety of DDEA 1.16% gel has been established in a number of settings, including osteoarthritis of the knee, neck pain, and ankle sprain [6–8]. DDEA 2.32% gel has shown efficacy and safety for treatment of ankle sprain [9]. To date, however, there have been no studies comparing the efficacy and safety of DDEA 1.16% gel and DDEA 2.32% gel. The objective of this study was to establish the noninferiority of DDEA 2.32% gel twice daily (BID) compared with DDEA 1.16% gel four times daily (QID).

## Methods

### Study design and patients

This was a phase 3, randomized, double-blind, multicenter, active-controlled, parallel-group, noninferiority study conducted at 15 sites in China from October 2019 to November 2020 (ClinicalTrials.gov registry number NCT04052620, 12 August 2019). It was designed to evaluate the efficacy and safety of DDEA 2.32% gel BID compared with DDEA 1.16% gel QID in patients with acute ankle sprain. The primary objective was to demonstrate noninferiority between DDEA 2.32% gel BID and DDEA 1.16% gel QID on day 5. Patients were randomized 1:1 to receive DDEA 2.32% gel applied BID or DDEA 1.16% gel applied QID. Patients in the BID group also received placebo BID so that all participants had 4 daily topical applications. Trial investigators enrolled patients, and a Centralized Randomization Center using an Interactive Response Technology was used to randomly allocate participants to treatment groups. Participants, care providers, investigators, and outcomes assessors were blinded.

The final study protocol and amendments, informed consent, and other relevant information were approved by ethics committees at each of the following 15 participating sites in accordance with China’s good clinical practice and other applicable China-specific requirements:

- Ruijin Hospital Ethics Committee
- Ethics Committee of Shanghai General Hospital Institutional Review Board
- Shanghai East Hospital Ethics Committee
- Ethics Committee of The No. 920 Hospital Ethics Committee of the Joint Logistic Support
- Force of the People’s Liberation Army
- Shanghai Fengxian District Central Hospital Ethics Committee
- Shanghai Pudong Hospital Ethics Committee
- Ethics Committee of Affiliated Zhongshan Hospital of Dalian University
- Beijing Pinggu Hospital Ethics Committee
- Ethics Committee of Chenzhou First People’s Hospital
- Ethics Committee of The Third Hospital of Hebei Medical University
- Ethics Committee of The First Affiliated Hospital of Xi’an Jiaotong University
- Medical Ethics Committee of Shenzhen Second People’s Hospital
- Medical Ethics Committee of The University of Hong Kong - Shenzhen Hospital
- Shenyang Orthopedic Hospital Ethics Committee
- Ethics Committee of The First Affiliated Hospital of Jinan University

The study was conducted in accordance with International Conference on Harmonisation and the ethical principles of the Declaration of Helsinki. Written, informed consent was obtained from each subject prior to any study-specific procedures.

Eligible patients were 18 to 75 years of age, had experienced an acute grade I–II sprain of the ankle within the past 24 hours, experienced pain on movement (POM) of at least 50 mm on a 100-mm visual analogue scale (VAS), and received no pain medication within 24 hours prior to randomization (RICE treatment prior to randomization was permitted). Stable daily doses of aspirin (≤162 mg/day) for at least 30 days prior to the first dose of study medication for nonanalgesic reasons were permitted and were continued for the study duration. Patients with a prior grade I–III sprain of the affected ankle within 3 months, a prior grade II–III sprain, other significant injury, or surgery of the affected ankle were excluded. Other exclusion criteria included pain or instability of the affected ankle due to a prior ankle sprain, ankle sprain attributable to a known disease affecting the ligaments, any skin lesion or wound in the area to be treated, and a plan to undergo surgery during the time of study participation. Use of concomitant systemic or topical NSAIDs, steroids (injected or oral), physiotherapy or other kind of pain therapy, tranquilizers, anxiolytics, hypnotics, sedatives, amphetamines, barbiturates, benzodiazepines, cocaine, methamphetamines, opiates, phencyclidine, tetrahydrocannabinol, and traditional herbal or homeopathic treatments were not permitted during the study. Other background maintenance medications that were not specifically excluded as described above were allowed during the study, provided the patient had been stable on the medications for at least 3 months. Adhesive and/or immobilizing casts, bandages, air splints, and RICE treatment were not allowed. Use of a crutch and certain physician-approved exercises were allowed. All patients were provided with a supply of acetaminophen (500 mg) as rescue therapy.

### Endpoints and assessments

The primary efficacy endpoint was the change from baseline in POM on day 5 of treatment as assessed by the patient using a 100-mm VAS. POM assessments were also conducted on days 1, 3, and 8 (±1 day). The investigator assessed POM while performing a manipulation of the ankle as the subject lay on an even horizontal surface.

Secondary efficacy variables included tenderness measured using calibrated algometers (pressure pain meter) in an area of 1 cm^2^ at the center of the injured area [10]; the patient indicated with a verbal cue when the onset of pain occurred. Other secondary endpoints included measures of ankle joint function using the Karlsson Scoring Scale [11, 12] and ankle swelling using the Figure of Eight Method [13]. Patients also completed a pain diary, which included pain intensity measured on a 4-point scale (0 = no pain to 3 = severe pain) and spontaneous pain relief assessed on a 5-point scale (0 = no relief to 4 = complete relief). Pain intensity and spontaneous pain relief were assessed immediately prior to the first dose and every 2 hours (±30 minutes) on day 1 and day 5 until the patient went to bed. Adverse event (AE) data were collected throughout the study and for 28 days following the last administration of study drug.

### Statistical analysis

Approximately 300 patients were planned to be randomized to ensure that at least 240 evaluable patients completed the study for the per protocol (PP) population. Sample size calculations determined that approximately 120 patients per treatment arm provided 80% power to demonstrate the noninferiority of DDEA 2.32% gel BID versus DDEA 1.16% gel QID by comparing the 2-sided 95% confidence interval (CI) of the difference in mean change from baseline of the primary endpoint of VAS POM between the 2 doses with a noninferiority margin of 13 mm. This noninferiority margin was chosen based on minimally important clinical differences reported in the literature [14]. This assumed a treatment standard deviation (SD) of 22 mm and allows for a possible small true treatment difference of 5 mm in favor of DDEA 1.16% gel QID.

To ensure high similarity between treatment arms, efficacy endpoints were analyzed using the PP population, defined as all randomized patients who had no major protocol deviations and had at least 1 post-baseline POM VAS assessment. The primary noninferiority endpoint of the change from baseline in POM for DDEA 2.32% gel BID versus DDEA 1.16% gel QID at day 5 was analyzed using an analysis of covariance (ANCOVA) model with treatment and center as factors and baseline POM as a covariate to estimate the treatment difference and 2-sided 95% CI without imputation of missing data. Secondary efficacy assessments were analyzed using an ANCOVA model including treatment arm and center as main effects, and baseline values as covariates, with treatment difference and 2-sided 95% CI presented for each assessment.

Pain intensity was analyzed based on Sum of Pain Intensity Difference (SPID) scores, defined from 0 to 24 hours or 0 to 12 hours after the first dose on day 1 and 0 to 24 hours or 0 to 12 hours after the first dose on day 5. Pain relief was analyzed based on the sum of total pain relief scores (TOTPAR), defined as from 0 to 24 hours or 0 to 12 hours after the first dose on day 1 and 0 to 24 hours or 0 to 12 hours after the first dose on day 5.

Use of rescue medication was compared between treatments using the Cochran-Mantel-Haenszel method stratified by center, with the median treatment differences (2-sided 95% CI) presented using Hodges-Lehmann estimation. AEs were analyzed using the safety population, defined as all patients who received at least 1 dose of study treatment. AEs were mapped to system-organ class and preferred term using MedDRA version 23.0.

## Results

### Patients

**Fig. 1:**
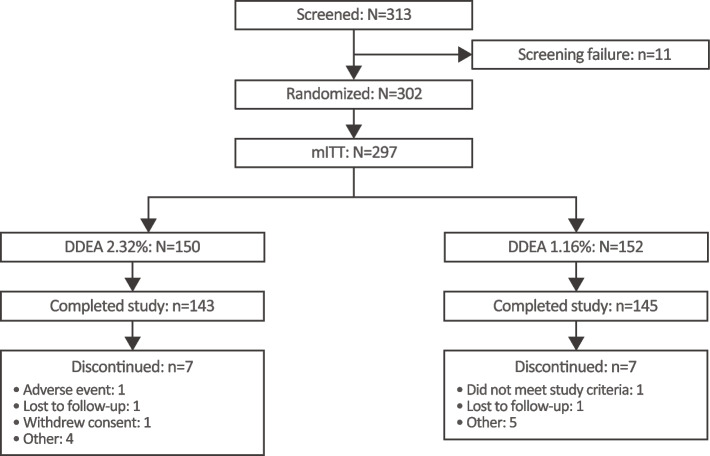
Patient disposition. DDEA, diclofenac diethylamine

A total of 313 patients were screened and 302 were randomized (intent-to-treat [ITT] population) at 15 sites in China from October 2019 to November 2020. The safety population comprised 301 patients, the modified ITT (mITT, all patients who had at least one postbaseline POM VAS assessment) 297 patients, and the PP population 250 patients. Overall, 95.4% of randomized patients completed the study, with 7 patients discontinuing treatment in each arm (Fig. 1). Patient demographics and baseline characteristics were well balanced between arms (Table 1). The mean age was 34 years, and 56% of patients were male; all patients were Asian. The majority of patients (63.2%) had a grade I ankle sprain with a partial tear of the ligament and 36.8% had a grade II sprain with an incomplete tear of the ligament and moderate functional impairment.

**Table 1:** Patient demographics and baseline characteristics

	DDEA 2.32% gel BID (***N*** = 150)	DDEA 1.16% gel QID (***N*** = 152)
Mean age, years (SD)	34.0 (11.1)	34.1 (12.4)
Median age, years (range)	32.0 (18, 67)	31.0 (18, 70)
Gender, n (%)		
Male	86 (57.3)	83 (54.6)
Female	64 (42.7)	69 (45.4)
Mean BMI, kg/m^2^ (SD)	23.8 (3.8)	24.2 (3.9)
Study ankle, n (%)		
Right	72 (48.0)	73 (48.0)
Left	78 (52.0)	79 (52.0)
Sprain classification, n (%)		
Grade I: partial tear of a ligament	94 (62.7)	97 (63.8)
Grade II: Incomplete tear of a ligament, with moderate functional impairment	56 (37.3)	55 (36.2)
POM by 100-mm VAS, mean (SD)	68.5 (10.7)	67.2 (9.7)
Tenderness by algometry, N/cm^2^		
Mean (SD)	18.0 (13.3)	16.6 (11.5)
Median (range)	14.7 (0.2, 85.4)	13.7 (1.7, 67.4)
Karlsson Score total score		
Mean (SD)	27.6 (17.4)	26.5 (15.8)
Median (range)	26.0 (0, 70.0)	25.0 (0, 70.0)
Figure of Eight Method		
Mean (SD)	53.2 (4.58)	52.6 (4.24)
Median (range)	52.5 (43.3, 70.0)	52.5 (43.2, 66.5)

*BID* twice daily, *BMI* body mass index, *DDEA* diclofenac diethylamine, *POM* pain on movement, *QID* four times daily, *SD* standard deviation, *VAS* visual analogue scale

### Primary endpoint

The mean (SD) change in POM from baseline to day 5 using the 100-mm VAS was − 42.8 mm (19.7 mm) with DDEA 2.32% gel BID and − 43.1 mm (18.1 mm) with DDEA 1.16% gel QID for the PP population (noninferiority margin 13 mm). The least squares mean difference (DDEA gel 2.32% – DDEA gel 1.16%) (mm) at this timepoint was 1.11 mm (95% CI − 3.00, 5.22; *P* = 0.595), and the upper limit (5.22 mm) of the 95% CI was less than the noninferiority margin of 13 mm, demonstrating that DDEA 2.32% gel BID was noninferior to DDEA 1.16% gel QID.

### Secondary endpoints

**Fig. 2:**
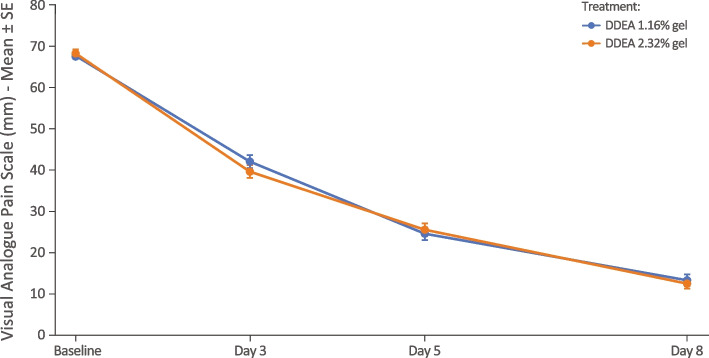
Mean (SE) for pain on movement by 100-mm VAS by visit (PP population). DDEA, diclofenac diethylamine; PP, per protocol; SE, standard error; VAS, visual analogue scale

The mean change from baseline for POM across the whole study period is shown in Fig. 2. There were no statistically significant differences between the 2 arms at any timepoint, with the least square mean difference on days 3 and 8 being − 2.43 mm (95% CI − 6.61, 1.75) and − 0.76 mm (− 4.23, 2.70), respectively, indicating that DDEA 2.32% gel BID was noninferior to DDEA 1.16% gel QID over the whole study period.

As shown in Table 2, there were no significant differences between the 2 treatments at any timepoint for tenderness measured by algometry and ankle joint function measured by the Karlsson Scoring Scale. Inflammation/swelling, tenderness, and joint function also improved at days 3, 5, and 8 with both formulations. However, there were statistically significant improvements in swelling of the ankle joint as measured using the Figure of Eight Method on days 5 and 8 with DDEA 2.32% gel BID compared with DDEA 1.16% gel QID (Table 2).

**Table 2:** Change from baseline in objective measures of affected ankle tenderness, joint function, and swelling

Least squares mean (SE)	DDEA 2.32% gel BID (***N*** = 122)	DDEA 1.16% gel QID (***N*** = 128)	Least squares mean difference (95% CI)^**a**^
Algometry, N/cm^2^			
Day 3	6.92 (0.85)	5.73 (0.83)	1.20 (−1.13, 3.54)
Day 5	9.84 (0.97)	10.76 (0.94)	−0.93 (−3.60, 1.74)
Day 8	17.06 (1.19)	15.60 (1.16)	1.46 (−1.83, 4.74)
Karlsson Scoring			
Day 3	16.26 (1.07)	15.52 (1.05)	0.74 (−2.23, 3.71)
Day 5	27.94 (1.46)	28.22 (1.43)	−0.28 (−4.33, 3.77)
Day 8	41.87 (1.59)	39.75 (1.56)	2.12 (−2.29, 6.54)
Figure of Eight Method			
Day 3	−0.42 (0.09)	−0.24 (0.09)	−0.18 (− 0.44, 0.07)
Day 5	−0.97 (0.10)	−0.57 (0.10)	−0.39 (− 0.66, − 0.12)
Day 8	−1.25 (0.10)	−0.87 (0.10)	−0.38 (− 0.66, − 0.10)

*BID* twice daily, *CI* confidence interval, *DDEA* diclofenac diethylamine, *QID* four times daily, *SE* standard error

^a^Confidence intervals encompass zero, indicating no significant difference between groups

**Fig. 3:**
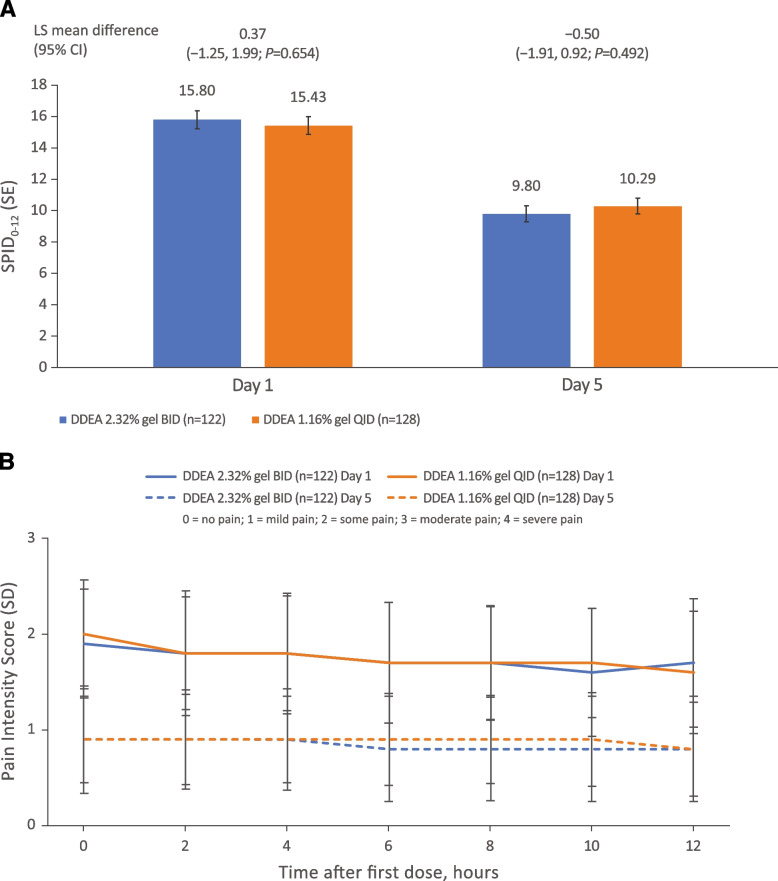
Pain intensity scores. **A** Sum of pain intensity difference over 12-hour interval after first dose (SPID_0–12_) on days 1 and 5. **B** Pain intensity scores at different timepoints following administration of first dose on days 1 and 5. Pain intensity was recorded in a patient diary and measured on a 4-point scale (0 = no pain to 3 = severe pain). BID, twice daily; CI, confidence interval; DDEA, diclofenac diethylamine; LS, least squares; QID, four times daily; SD, standard deviation; SE, standard error; SPID_0–12_, sum of pain intensity difference scores from 0 to 12 hours following first dose

**Fig. 4:**
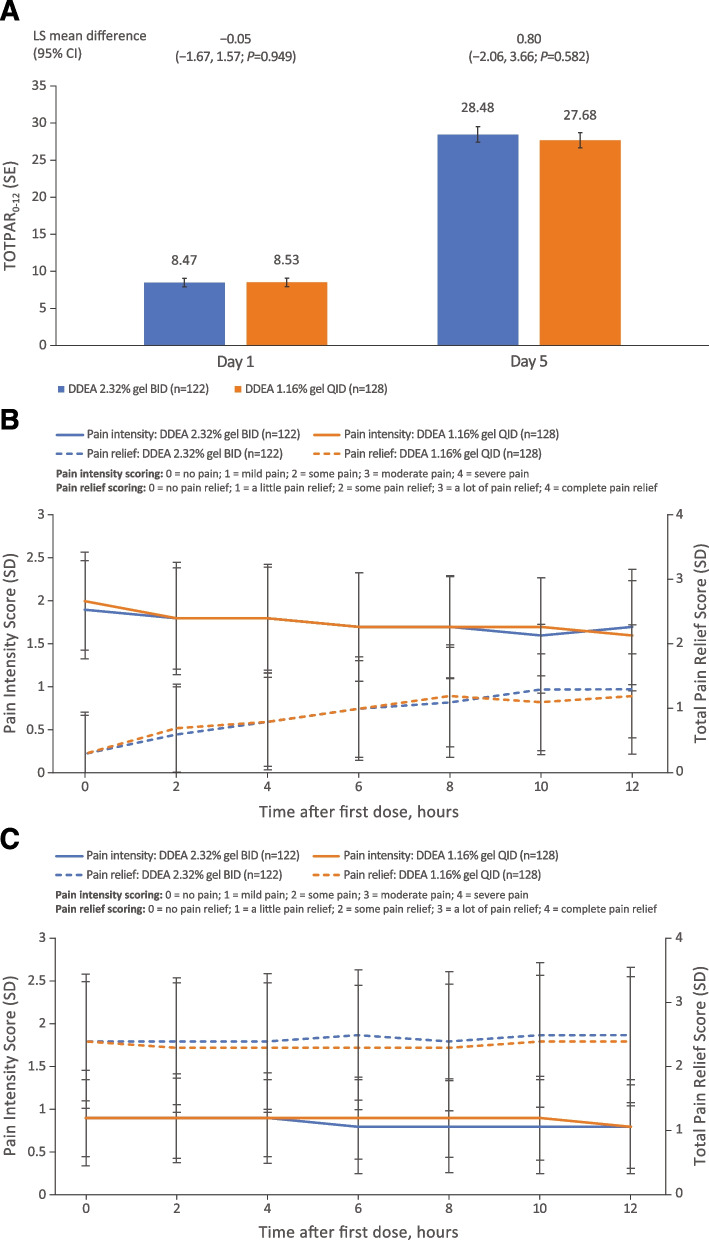
Total pain relief. **A** Sum of total pain relief scores over 12-hour interval following first dose (TOTPAR_0–12_) on days 1 and 5. **B** Pain intensity and pain relief scores on day 1. **C** Pain intensity and pain relief scores on day 5. Pain relief was recorded in a patient diary and assessed on a 5-point scale (0 = no relief to 4 = complete relief). BID, twice daily; CI, confidence interval; DDEA, diclofenac diethylamine; LS, least squares; QID, four times daily; SD, standard deviation; SE, standard error; TOTPAR_0–12_, sum of total pain relief scores from 0 to 12 hours after first dose

Based on patient pain diaries, there was no significant difference between the 2 formulations in terms of the time-weighted sum of SPID in the 12 hours following the first dose on day 1 and the first dose on day 5 (Fig. 3A). Furthermore, both formulations demonstrated reduced pain intensity following treatment on day 5 compared with day 1; there was no significant difference between treatments in intensity at any time point on days 1 and 5 (Fig. 3B). Both formulations also provided notable increases in pain relief from day 1 to day 5. There was no significant difference between DDEA 2.32% gel BID and DDEA 1.16% gel QID in terms of the time-weighted sum of TOTPAR during the 0- to 12-hour interval following the first dose on day 1 and the first dose on day 5 (Fig. 4A) or at any individual timepoint over the 12 hours following the first dose on days 1 or 5 (Fig. 4B). Pain relief was sustained over 12 hours for both treatment arms on day 5. Notably, for DDEA 2.32% gel, pain relief at hour 0 of day 5 started from a higher index (2.4, right axis of Fig. 4C) than that at hour 12 of day 1 (1.3, right axis of Fig. 4B), and pain relief scores remained steady throughout the 12-hour interval on day 5. Pain intensity at hour 0 of day 5 started from lower index (0.9, left axis of Fig. 4C) than that at hour 12 of day 1 (1.7, left axis of Fig. 4B), and pain intensity remained steady throughout the 12-hour interval on day 5. From day 1 to day 5, pain decreased and pain relief increased after treatment (Fig. 4B and C). Similar trends were observed in the DDEA 1.16% gel arm.

Eight patients (5.3%) in the DDEA 2.32% gel BID arm and 12 patients (7.9%) in the DDEA 1.16% gel QID arm took rescue medication. The mean (SD) number of rescue acetaminophen tablets taken was 2.4 (2.4) and 2.6 (2.2) in the DDEA 2.32% gel BID and DDEA 1.16% gel QID arms, respectively. The primary reasons for rescue medication (self-administered) were headache, stomachache, and toothache.

### Safety

There was no significant difference in the incidence of treatment-emergent AEs (TEAEs) or AEs adjudicated as being treatment related. In the DDEA 2.32% gel BID arm, 11 patients experienced 13 AEs, with 3 patients experiencing 3 treatment-related AEs. Similarly, in the DDEA 1.16% gel QID arm, 12 patients experienced 13 AEs, with 2 patients experiencing 3 treatment-related AEs. There were no serious TEAEs, and no single AE occurred in more than 2 patients in either arm. Treatment-related AEs were application site inflammation (*n* = 1), dermatitis allergic (*n* = 1), and skin exfoliation (*n* = 1) in 3 patients in the DDEA 2.32% gel BID arm, and arthralgia (*n* = 1), dermatitis allergic (*n* = 1), and rash (*n* = 1) in 2 patients in the DDEA 1.16% gel QID arm. One patient discontinued from the DDEA 2.32% gel BID arm due to application-site inflammation. Incidence rates and types of TEAEs were consistent with DDEA labeling [15].

## Discussion

This study demonstrates that DDEA 2.32% gel BID is noninferior to DDEA 1.16% gel QID as measured by the mean change from baseline in POM at day 5 following an ankle sprain. There also was no difference between DDEA 2.32% gel BID and DDEA 1.16% gel QID in terms of tenderness, ankle joint function, and patient-reported measures of pain intensity and pain relief. Furthermore, DDEA 2.32% gel BID conferred 12-hour pain relief (TOTPAR_0–12_) and reduction in pain intensity (SPID_0–12_) similar to that of DDEA 1.16% gel QID. There was a statistically significant difference between groups for swelling favoring DDEA 2.32% gel BID on days 5 and 8.

Both formulations of DDEA gel markedly improved POM, ankle tenderness, joint functioning, and swelling of the affected ankle. For POM, these changes began as early as day 1, became clinically relevant on day 3, and continued to improve on days 5 and 8. There were similar improvements over time for tenderness, joint function, and swelling. Furthermore, pain relief was sustained over 12 hours for both treatment arms on day 5.

In the legacy study (VOPO-P-307) [9], the mean (SD) change in POM from baseline to day 3 with DDEA 2.32% gel BID was − 32.4 mm (19.6 mm). Furthermore, at day 3, DDEA 2.32% gel BID demonstrated significantly superior pain relief versus placebo as measured by the difference in reduction of POM (− 14.1 mm; 95% CI − 18.8 to − 9.4; *P* < 0.0001) [9]. In the current study, the mean change in POM from baseline to day 3 with DDEA 2.32% gel BID was − 28.7 mm (SD 19.37 mm), which is similar to the values reported in the legacy study, supporting the assertation that DDEA 2.32% demonstrates pain relief at day 3.

A limitation of this study is that it was not placebo controlled. However, the results are validated and supported by extensive data demonstrating the effectiveness of DDEA 1.16% gel QID and DDEA 2.32% gel BID [6–9]. A systematic Cochrane review including all formulations of topical diclofenac further confirmed its efficacy, finding a risk ratio for clinical success of 1.60 (95% CI 1.49, 1.72) favoring diclofenac over placebo [16]. Furthermore, a pharmacokinetic bioequivalence study conducted in 40 healthy volunteers demonstrated equivalent systemic absorption for DDEA 1.16% QID and DDEA 2.32% BID (unpublished data), indicating that application frequency does not affect systemic absorption and supporting noninferiority for efficacy. Furthermore, it is important to note that systemic exposure with topical diclofenac formulations is approximately 50-fold lower than with oral administration, so clinically relevant systemic exposure is not anticipated. As a result, systemic side effects are anticipated to be negligible and not clinically significant [17]. Consistent with this, there were no apparent systemic side effects, and most AEs considered to be treatment-related were dermatologic at the site of administration. Furthermore, there was no difference in treatment-related AEs between DDEA 1.16% gel QID and DDEA 2.32% gel BID, and AEs were consistent with current labeling for DDEA gel.

Treatment adherence in the context of this clinical trial was good, with 91 and 88% of patients in the DDEA 2.32% gel BID and DDEA 1.16% gel QID arms, respectively, having good adherence with study medications. In a real-world setting, the added convenience of BID versus QID administration should increase treatment adherence, resulting in better efficacy and patient satisfaction [9].

## Conclusions

DDEA 2.32% gel BID offers similar pain reduction and relief, anti-inflammatory effects, and tolerability as DDEA 1.16% gel QID. DDEA 2.32% gel BID offers a convenient alternative to DDEA 1.16% gel QID.

## Data Availability

The aggregated data that support the findings of this study are available upon reasonable request from GSK Consumer Healthcare S.A. Requests must include a research proposal describing the objectives of research and its benefits for patients accompanied by a sufficient description of statistical and publication plans. Each request will be reviewed on an individual basis by GSK Consumer Healthcare to assess the ability of the proposal to meet the proposed scientific objectives and relevance to patient care.

## Abbreviations

AE: Adverse event
ANCOVA: Analysis of covariance
BID: Twice-daily
BMI: Body mass index
CI: Confidence interval
COX-1: Cyclooxygenase 1
COX-2: Cyclooxygenase 2
DDEA: Diclofenac diethylamine
ITT: Intent to treat
LS: Least squares
MedDRA: Medical Dictionary for Regulatory Activities
NSAID: Nonsteroidal anti-inflammatory drugs
POM: Pain on movement
PP: Per protocol
QID: Four-times-daily
SD: Standard deviation
SE: Standard error
SPID: Sum of Pain Intensity Difference
SPID_0–12_: Sum of pain intensity difference scores from 0 to 12 hours following first dose
TOTPAR: Total pain relief scores
VAS: Visual analogue scale
